# The risk of inappropriate empiric treatment and its outcomes based on pathogens in non-ventilated (nvHABP), ventilated (vHABP) hospital-acquired and ventilator-associated (VABP) bacterial pneumonia in the US, 2012–2019

**DOI:** 10.1186/s12879-022-07755-y

**Published:** 2022-10-05

**Authors:** Marya D. Zilberberg, Brian H. Nathanson, Laura A. Puzniak, Ryan J. Dillon, Andrew F. Shorr

**Affiliations:** 1 EviMed Research Group, LLC, P.O. Box 303, Goshen, MA 01032 USA; 2 OptiStatim, LLC, Longmeadow, MA USA; 3 Merck & Co., Inc., Rahway, NJ USA; 4 Washington Hospital Center, 110 Irving St. NW, Washington, DC 20010 USA

**Keywords:** Pneumonia, Noosocomial pneumonia, Hospital-acquired pneumonia, Outcomes

## Abstract

**Background:**

Inappropriate empiric antimicrobial treatment (IET) contributes to worsened outcomes. While IET’s differential impact across types of nosocomial pneumonia (NP: non-ventilated [nvHABP], ventilated [vHABP] hospital-acquired and ventilator-associated [VABP] bacterial pneumonia) is established, its potential interaction with the bacterial etiology is less clear.

**Methods:**

We conducted a multicenter retrospective cohort study in the Premier Healthcare Database using an administrative algorithm to identify NP. We paired respective pathogens with empiric treatments. Antimicrobial coverage was appropriate if a drug administered within 2 days of infection onset covered the recovered organism(s). All other treatment was IET.

**Results:**

Among 17,819 patients with NP, 26.5% had nvHABP, 25.6% vHABP, and 47.9% VABP. Gram-negative (GN) organisms accounted for > 50% of all infections. GN pathogens were ~ 2 × as likely (7.4% vHABP to 10.7% nvHABP) to engender IET than Gram-positive (GP, 2.9% vHABP to 4.9% nvHABP) pathogens. Although rare (5.6% nvHABP to 8.3% VABP), GN + GP infections had the highest rates of IET (6.7% vHABP to 12.9% nvHABP). Carbapenem-resistant GNs were highly likely to receive IET (33.8% nvHABP to 40.2% VABP). Hospital mortality trended higher in the IET group, reaching statistical significance in GN + GP vHABP (47.8% IET vs. 29.3% non-IET, p = 0.016). 30-day readmission was more common with IET (16.0%) than non-IET (12.6%, p = 0.024) in GN VABP. Generally post-infection onset hospital length of stay and costs were higher with IET than non-IET.

**Conclusions:**

IET is ~ 2 × more common in GN than GP infections. Although the magnitude of its impact varies by NP type, IET contributes to worsened clinical and economic outcomes.

**Supplementary Information:**

The online version contains supplementary material available at 10.1186/s12879-022-07755-y.

## Background

Nosocomial pneumonia (NP) remains a persistent clinical problem fraught with both high hospital mortality and costs [1, 2]. Although most accept that hospital-acquired (HABP) and ventilator-associated (VABP) bacterial pneumonias are substantively different from each other, recent findings suggest that HABP rubric fails to capture significant distinctions between patients with HABP who do and those who do not require mechanical ventilation (MV) to manage this infection. Indeed, HABP requiring MV (vHABP) and not requiring MV (nvHABP) differ along patient characteristics, pathogen distributions, and hospital outcomes [3–7]. For example, while the rates of both *P. aeruginosa* and methicillin-resistant *S. aureus* (MRSA) are higher among patients with nvHABP, the prevalence of *E. coli* and methicillin-susceptible *S. aureus* (MSSA) in vHABP exceeds that in nvHABP [6]. Most strikingly, hospital mortality and costs are substantially higher in vHABP [5].

One key challenge in all forms of NP is reducing the risk of administering inappropriate empiric therapy (IET). Though a plethora of studies underscore the detrimental impact of IET on outcomes, instituting correct empiric treatment remains a challenge. This is mostly due to two factors: (1) most institutions continue to rely on culture technology, which takes 2–3 days to return antibacterial susceptibility results, and (2) a shifting landscape of antimicrobial resistance (AMR) [8]. This means that clinicians are often forced to treat serious infections based on suboptimal risk stratification schemes, formal or informal, to address the pathogen(s) patients are most likely to harbor. Hence, a certain proportion of patients are exposed to antimicrobials that do not cover their infection, and thus face an increased risk of mortality and further morbidity [8]. Furthermore, just as patient and pathogen characteristics differ, the rates and consequences of IET in NP are also not uniform across its types. Although we have noted that the rates of IET in NP are lower than previously reported, there is a gradient of risk, lowest in vHABP (5.6%) to highest in nvHABP (8.5%) [6]. Commensurately, attributable hospital post-infection onset length of stay (LOS) and costs are nearly 10 times and three times, respectively, greater in nvHABP than in vHABP, and three times and twice, respectively, higher in nvHABP than in VABP [7].

Because studies that utilize modeling to understand the impact of IET on outcomes may miss subtle but important differences within various strata of infections, despite exploring interaction terms, it is important to examine these patterns within more homogeneous groups of patients. More specifically, it is not well understood whether and how specific pathogens interact with the risk of IET exposure and consequent associated outcomes. For example, IET for MRSA in VABP may carry different implications than IET for *P. aeruginosa* in nvHABP. Understanding these subtleties with greater granularity could help clinicians to target their treatment to most common and significant pathogens that prevail in their institutions. Therefore, we examined the risk of IET across the most common bacterial pathogens isolated from patients with nvHABP, vHABP, and VABP, and its impact on pathogen- and pneumonia-type-specific outcomes.

## Methods

### Ethics statement

Because this study used already existing fully de-identified data, it was exempt from ethics review under US 45 CFR 46.101(b)4 [9].

### Study design and patient population

This was a multi-center retrospective cohort study of hospitalized patients with culture-positive nvHABP, vHABP, or VABP. The case identification approach relied on a published administrative algorithm [10]. The details of the study methods can be found in citations #5, #6, and #7, all analyses conducted in the same cohort [5–7]. We included adults (age ≥ 18 years) with a pneumonia diagnosis in a secondary position, with an index respiratory and/or blood culture obtained on hospital day 3 or later for HABP, or on MV day 3 or later for VABP, and with evidence of antibiotic treatment on the day of the index culture and for the next ≥ 3 consecutive days. To reduce misclassification, patients fitting the definition for a complicated urinary tract infection or a complicated intra-abdominal infection were excluded [11, 12].

### Data source

The data derived from the Premier Research database, an electronic laboratory, pharmacy and billing data repository, for years 2012 through the 3rd quarter of 2019. For a detailed description of the database, please, see citations [5–7, 10–15]. Approximately 200 US institutions submitted microbiology data during the study time frame. Further details of the current cohort have been published elsewhere [5–7].

### Pneumonia classification

HABP was defined as a pneumonia with the index culture occurring with the patient not on MV, and VABP with the index culture during MV for 3 + days. HABP was further categorized into vHABP and nvHABP. Namely, we defined vHABP in patients who needed MV ≤ 5 days following the onset of the index HABP episode; conversely, HABP was nvHABP when MV was not required within the same time frame.

### Microbiology and empiric treatment

We examined pathogens that commonly cause bacterial nosocomial pneumonia. Specific organisms of interest were *Pseudomonas aeruginosa, Acinetobacter baumannii*, *Stenotrophomonas maltophilia, Haemophilus spp, Moraxella catarrhalis, Enterobacterales*, *Staphylococcus aureus* (both MSSA and MRSA), *Streptococcus pneumoniae*, and other *Streptococcus spp*.

Susceptibilities were grouped into S (susceptible) or R (resistant, combining both R and intermediate, I). Generally, carbapenem resistance (CR) required a gram-negative organism to be I or R to imipenem, meropenem, ertapenem, or doripenem. CR in *P. aeruginosa* and *A. baumannii* was defined more narrowly as I or R to anti-pseudomonal carbapenems—imipenem, meropenem, or doripenem. All *S. maltophilia* isolates were assumed to be CR. Susceptibility to either a carbapenem or to a third-generation cephalosporin signified carbapenem susceptibility (CS). Non-susceptibility among *Enterobacterales* to any one of the third generation cephalosporins (C3R) was examined as a phenotypic surrogate for extended spectrum beta-lactamase (ESBL) containing organisms. Finally, we identified pathogens resistant to the following non-carbapenem anti-pseudomonal beta lactams: cefepime, ceftazidime, ceftazidime/avibactam, ceftolozane/tazobactam, and piperacillin/tazobactam.

Appropriate empiric coverage was a drug that covered the identified pathogen and was administered within 2 days of the index culture being obtained. Appropriateness of treatment was considered “indeterminate” if there were not adequate susceptibility data either reported directly or subject to the above algorithms. All other treatment was considered IET.

All microbiology results were based on the local testing done by participating hospitals using CLSI breakpoints to determine susceptibilities.

### Outcome variables

The primary outcome of interest was all-cause hospital mortality. Secondary outcomes included post-infection onset MV duration; post-infection ICU LOS; hospital LOS (total and post-infection onset); hospital costs; and 30-day readmission rates among survivors.

### Statistical analyses

We report descriptive statistics to compare the rates of IET for every pathogen of interest stratified by pneumonia type. We further grouped the pathogens by pertinent susceptibility profiles and whether they were Gram-negative or Gram-positive to derive similar comparisons. Finally, we explored the outcomes in nvHABP, vHABP, and VABP groups stratified by both Gram-stain category and IET.

Continuous variables are reported as means with standard deviations (SD) and as medians with interquartile ranges (IQR). Differences between mean values were tested via a one-way ANOVA test, and between medians using the Kruskal–Wallis test. Categorical data are summarized as proportions, with the Chi-square test used to examine inter-group differences unless a cell count was < 5, wherein the Fisher exact test was used. p-values < 0.05 were considered statistically significant.

## Results

Among 17,819 patients who met enrollment criteria, 26.5% had nvHABP, 25.6% vHABP, and 47.9% VABP. Gram-negative (GN) pathogens accounted for > 50% of all infections across NP types (Table 1). IET was administered to 8.5% in nvHABP, 5.6% in vHABP, and 7.2% in VABP. Patients with a GN pathogen were ~ 2 × as likely (7.4% vHABP to 10.7% nvHABP) to receive IET than persons with a Gram-positive (GP, 2.9% vHABP to 4.9% nvHABP) pathogen. Individuals with mixed GN + GP infections had the highest rates of IET (6.7% vHABP to 12.9% nvHABP). Both C3R (up to 24.3%, in nvHABP) and CR (up to 40.2%, in VABP) organisms placed patients at a higher risk for IET (Table 1).

**Table 1:** Pathogen characteristics and inappropriate empiric treatment

	nvHABP	%	vHABP	%	VABP	%	P-value
	N = 4728 (26.53%)		N = 4561 (25.60%)		N = 8530 (47.87%)		
Gram stain status							
GN only	2603	55.05%	2435	53.39%	4835	56.68%	
IET	278	10.68%	180	7.39%	449	9.29%	0.001
Indeterminate	200	7.68%	168	6.90%	329	6.80%	
Non-IET	2125	81.64%	2087	85.71%	4057	83.91%	
GP only	1862	39.38%	1781	39.05%	2987	35.02%	
IET	91	4.89%	51	2.86%	117	3.92%	0.007
Indeterminate	204	10.96%	164	9.21%	291	9.74%	
Non-IET	1567	84.16%	1566	87.93%	2579	86.34%	
GN + GP	263	5.56%	345	7.56%	708	8.30%	
IET	34	12.93%	23	6.67%	49	6.92%	< 0.001
Indeterminate	65	24.71%	46	13.33%	117	16.53%	
Non-IET	164	62.36%	276	80.00%	542	76.55%	
Specific resistant pathogens							
CR (among GN patients)	213	7.43%	186	6.69%	493	8.89%	
IET	72	33.80%	68	36.56%	198	40.16%	0.310
Indeterminate	27	12.68%	16	8.60%	43	8.72%	
Non-IET	114	53.52%	102	54.84%	252	51.12%	
C3R (among *Enterobaterales*)	272	14.43%	284	14.90%	493	12.86%	
IET	66	24.26%	46	16.20%	86	17.44%	0.032
Indeterminate	16	5.88%	15	5.28%	42	8.52%	
Non-IET	190	69.85%	223	78.52%	365	74.04%	

nvHABP = non-ventilated HABP; vHABP = ventilated HABP; VABP = ventilator-associated bacterial pneumonia; MRSA = methicillin-resistant *S. aureus*; MSSA = methicillin-susceptible *S. aureus*; SD = standard deviation; IQR = interquartile range; IET = inappropriate empiric therapy; GP = Gram positive; GN = Gram-negative; CR = carbapenem resistant; C3R = resistant to 3rd generation cephalosporins

**Fig. 1:**
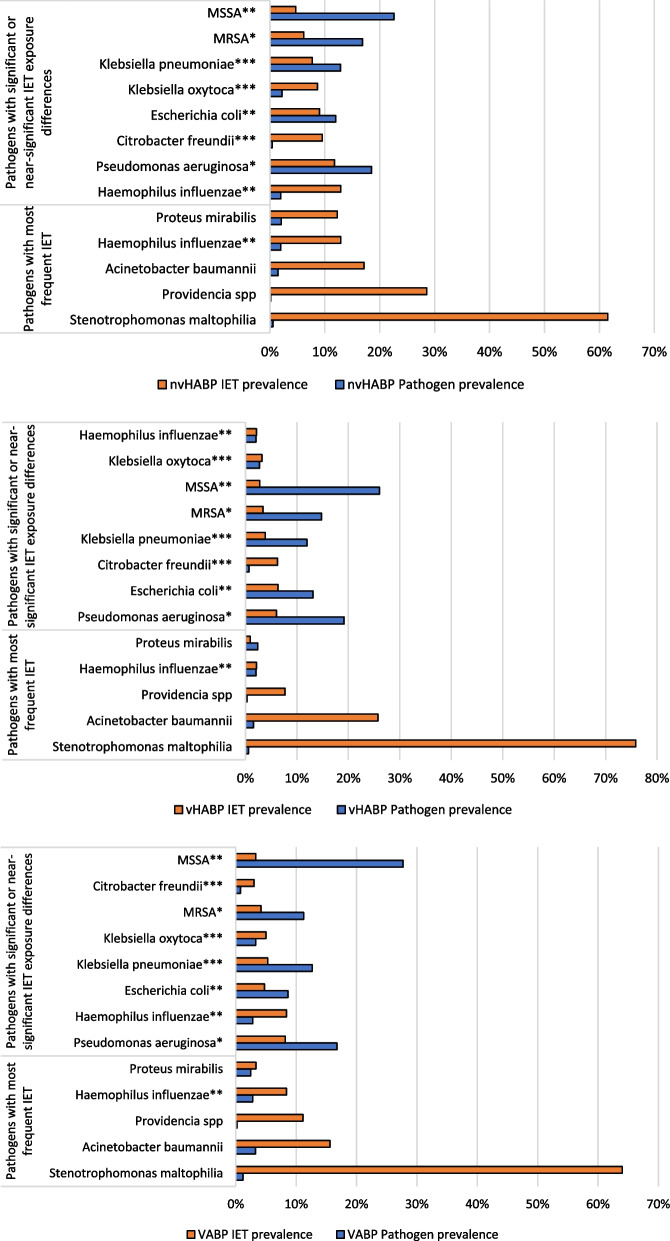
Pathogen and IET prevalence stratified by pneumonia type. *P < 0.001. **P < 0.050. ***P < 0.100. MSSA = methicillin-susceptible *S. aureus*; MRSA = methicillin-resistant *S. aureus*; nvHABP = non-ventilated hospital-acquired bacterial pneumonia; IET = inappropriate empiric treatment; vHABP = ventilated hospital-acquired bacterial pneumonia; VABP = ventilator-associated bacterial pneumonia

Across all pneumonia types, specific organisms with the most elevated risk for IET were *S. maltophilia*, *A. baumannii*, and *Providencia spp* (Additional file 1: Table S1). These isolates, however, were relatively infrequent. For example, *S. maltophilia* was isolated in 0.6% of nvHABP and vHABP, and 1.2% of VABP, while its IET risk ranged from 61.5% in nvHABP to 75.9% in vHABP (Figs. 1a–c). Indeed, among all patients receiving IET, ten organisms accounted for 88% of all IET administered within nvHABP, 89% within vHABP, and 86% within VABP groups (Table 2). However, among them, only the differences in IET prevalence across pneumonia types in MRSA, *P. aeruginosa*, MSSA, *H. influenzae*, and *E. coli* reached statistical significance (Additional file 1: Table S1).

**Table 2:** Pathogens most commonly accounting for IET exposure

	nvHABP		vHABP		VABP	
	N	%	N	%	N	%
*Pseudomonas aeruginosa*	103	24.18	45	17.37	117	18.51
*Escherichia coli*	51	11.97	38	14.67	35	5.54
*MSSA*	50	11.74	33	12.74	78	12.34
*MRSA*	49	11.50	23	8.88	40	6.33
*Klebsiella pneumoniae*	47	11.03	21	8.11	57	9.02
*Enterobacter cloacae*	21	4.93	20	7.72	59	9.34
*Stenotrophomonas maltophilia*	16	3.76	22	8.49	64	10.13
*Serratia marcescens*	15	3.52	11	4.25	42	6.65
*Proteus mirabilis*	12	2.82	1	0.39	7	1.11
*Acinetobacter baumannii*	12	2.82	17	6.56	43	6.80
*Other*	50	11.74	28	10.81	90	14.24

^*^"Other” category consists of the organisms listed in Additional file 2: Table S2

IET = inappropriate empiric treatment; nvHABP = non-ventilated hospital-acquired bacterial pneumonia; vHABP = ventilated hospital-acquired bacterial pneumonia; VABP = ventilated hospital-acquired bacterial pneumonia; MSSA = methicillin-susceptible *S. aureus*; MRSA = methicillin-resistant *S. aureus*

With the exception of mixed GN + GP infections, hospital mortality did not differ significantly between the IET and non-IET groups (Table 3). In the GN + GP group, mortality was numerically lower with IET in nvHABP and VABP, but significantly higher in vHABP (47.8% IET vs. 29.3% non-IET, p = 0.016) (Table 3). Similarly, 30-day readmission among those discharged alive, was significantly more common in the setting of IET (16.0%) than non-IET (12.6%, p = 0.024) in GN VABP only. The post-infection onset hospital length of stay was generally longer in the setting of IET, but did not reach statistical significance in every case (Table 3). For example, while the mean ± SD post-infection LOS among patients with a GN nvHABP was 20.1 ± 38.6 days with IET vs. 12.3 ± 18.9 days with non-IET (p < 0.001), in the setting of VABP this difference was much smaller, 21.5 ± 26.3 days with IET vs. 19.2 ± 23.9 days with non-IET, p = 0.141.

**Table 3:** Hospital outcomes*

	nvHABP		%	P-value	vHABP		%	P-value	VABP		%	P-value
	N = 4728				N = 4561				N = 8530			
	N received treatment	N with outcome	% with outcome		N received treatment	N with outcome	% with outcome		N received treatment	N with outcome	% with outcome	
Hospital mortality												
GN only												
IET	278	34	12.23%		180	61	33.89%		449	99	22.05%	
Indeterminate	200	28	14.00%	0.175	168	53	31.55%	0.423	329	77	23.40%	0.905
non-IET	2125	217	10.21%		2087	616	29.52%		4057	917	22.60%	
GP only												
IET	91	12	13.19%		51	15	29.41%		117	16	13.68%	
Indeterminate	204	17	8.33%	0.154	164	33	20.12%	0.057	291	45	15.46%	0.078
non-IET	1567	205	13.08%		1566	453	28.93%		2579	505	19.58%	
GP + GN (mixed)												
IET	34	4	11.76%		23	11	47.83%		49	6	12.24%	
Indeterminate	65	8	12.31%	0.555	46	7	15.22%	0.016	117	24	20.51%	0.211
non-IET	164	28	17.07%		276	81	29.35%		542	124	22.88%	
30-day readmission for survivors												
GN only												
IET	244	56	22.95%		119	23	19.33%		350	56	16.00%	
Indeterminate	172	32	18.60%	0.503	115	15	13.04%	0.304	252	44	17.46%	0.024
non-IET	1908	385	20.18%		1471	212	14.41%		3140	395	12.58%	
GP only												
IET	79	18	22.78%		36	10	27.78%		101	8	7.92%	
Indeterminate	187	26	13.90%	0.140	131	21	16.03%	0.148	246	33	13.41%	0.320
non-IET	1362	262	19.24%		1113	174	15.63%		2074	232	11.19%	
GP + GN (mixed)												
IET	30	7	23.33%		12	2	16.67%		43	6	13.95%	
Indeterminate	57	12	21.05%	0.931	39	7	17.95%	0.621	93	11	11.83%	0.836
non-IET	136	32	23.53%		195	26	13.33%		418	46	11.00%	
Post-infection onset LOS, days												
GN only												
IET												
Mean (SD)		20.1 (38.6)				20.0 (22.8)				21.5 (26.3)		
Median [IQR]		10 [6, 20]				15 [8, 23]				14 [8, 25]		
Indeterminate												
Mean (SD)		11.7 (15.2)		< 0.001		16.5 (16.9)		0.040		19.0 (18.8)		0.141
Median [IQR]		8 [4, 14]				11 [7, 12]				14 [8, 21]		
non-IET												
Mean (SD)		12.3 (18.9)				16.8 (15.6)				19.2 (23.9)		
Median [IQR]		8 [4, 14]				13 [7, 21]				14 [8, 23]		
GP only												
IET												
Mean (SD)		24.2 (40.9)				19.8 (18.3)				21.6 (18.5)		
Median [IQR]		13 [8, 20]				14 [10, 21]				16 [10, 26]		
Indeterminate												
Mean (SD)		12.3 (13.9)		< 0.001		17.5 (18.2)		0.531		17.5 (16.8)		0.079
Median [IQR]		7 [4, 15]				13 [7, 22]				13 [8, 22]		
non-IET												
Mean (SD)		12.9 (16.1)				16.9 (19.1)				17.9 (17.8)		
Median [IQR]		8 [5, 16]				13 [7, 20]				13 [7, 22]		
GP + GN (mixed)												
IET												
Mean (SD)		21.6 (20.7)				11.9 (8.0)				20.3 (23.3)		
Median [IQR]		13 [8, 25]				9 [6, 18]				14 [9, 22]		
Indeterminate												
Mean (SD)		11.5 (12.1)		0.002		22.2 (31.6)		0.037		21.6 (31.6)		0.146
Median [IQR]		8 [4, 14]				14 [10, 24]				14 [7, 24]		
non-IET												
Mean (SD)		12.7 (13.7)				15.7 (15.2)				17.8 (16.8)		
Median [IQR]		9 [5, 15]				12 [7, 20]				14 [8, 21]		
Hospital costs, $												
GN only												
IET												
Mean (SD)		76,777 (101,564)				102,478 (91,918)				123,268 (106,242)		
Median [IQR]		48,969 [29549, 86751]				77,245 [48008, 119903]				91,303 [56923, 148733]		
Indeterminate												
Mean (SD)		60,017 (56,357)		0.003		84,320 (71,074)		0.015		111,938 (88,926)		0.010
Median [IQR]		44,941 [28873, 71378]				65,476 [38497, 108844]				86,242 [56077, 137449]		
non-IET												
Mean (SD)		60,765 (70,877)				86,154 (72,384)				108,951 (94,676)		
Median [IQR]		41,468 [25678, 71369]				66,651 [41982, 105006]				86,242 [56077, 137449]		
GP only												
IET												
Mean (SD)		74,034 (91,243)				96,918 (102,939)				104,823 (86,049)		
Median [IQR]		42,942 [23666, 89352]				63,064 [53054, 106652]				84,941 [56245, 123236]		
Indeterminate												
Mean (SD)		53,716 (61,532)		0.005		71,290 (63,776)		0.211		84,858 (93,950)		0.344
Median [IQR]		39,149 [20891, 59493]				52,698 [32478, 87469]				59,201 [41028, 93012]		
non-IET												
Mean (SD)		53,122 (56,336)				76,731 (92,620)				88,807 (131,561)		
Median [IQR]		35,701 [22366, 62864]				57,132 [36363, 90331]				68,748 [46021, 106164]		
GP + GN (mixed)												
IET												
Mean (SD)		82,725 (69,808)				66,156 (44,002)				100,252 (73,853)		
Median [IQR]		56,469 [28881, 120956]				49,536 [33519, 103377]				73,872 [57637, 125257]		
Indeterminate												
Mean (SD)		53,461 (55,594)		0.014		83,150 (83,181)		0.609		95,257 (81,193)		0.445
Median [IQR]		37,407 [21347, 59886]				62,893 [40843, 89923]				72,527 [41122, 112679]		
non-IET												
Mean (SD)		56,488 (42,955)				76,610 (65,616)				89,641 (62,629)		
Median [IQR]		41,012 [26902, 73106]				57,327 [37997, 91906]				74,512 [48703, 115904]		

*All P values refer to the differences between IET, non-IET, and indeterminate within each pneumonia type and outcome

nvHABP = non-ventilated hospital-acquired bacterial pneumonia; vHABP = ventilated hospital-acquired bacterial pneumonia; VABP = ventilator-associated bacterial pneumonia; LOS = length of stay; SD = standard deviation; IQR = interquartile range; IET = inappropriate empiric therapy; GN = gram-negative; GP = gram-positive

Costs were also generally higher with IET than with non-IET, but not every comparison reached statistical significance (Table 3). Mean costs with IET significantly exceeded those with non-IET in all pneumonia types in the setting of a GN infection: nvHABP $76,777 ± $101,564 IET vs. $60,765 ± $70,877 non-IET, p = 0.003; vHABP $102,478 ± $91,918 IET vs. $86,154 ± $72,384 non-IET, p = 0.015, and VABP $123,268 ± $106,242 IET vs. $108,951 ± $94,676, p = 0.010. The same comparisons reached statistical significance only in nvHABP when the pathogen(s) was (were) GP (p = 0.005) or GN + GP (p = 0.014).

## Discussion

We demonstrate that, although less common than in past reports, IET continues to occur in the setting of NP, ranging in prevalence depending not only on pneumonia type, but also on the causative pathogen. While IET occurs in between 3 and 5% of only GP infections and between 7 and 11% of only GN ones, irrespective of pneumonia type the risk is much higher when there are both GN and GP pathogens present, reaching 13% in nvHABP. Likewise, specific organisms, such as *S. maltophilia*, e.g., though isolated in ~ 1% or fewer cases, pose a much greater risk of IET. This is consistent with the fact that AMR drives the rate of IET, ranging from 24% in C3R to 40% in CR organisms. More importantly, these patterns of IET carry different implications in different settings. Thus, hospital mortality increased with IET only among patients whose nvHABP is caused by a mix of GN and GP organisms, while 30-day readmission is reduced with non-IET only in GN VABP. While LOS and hospital costs are consistently numerically higher with IET in all pneumonia types, particularly when caused by a GN or GN + GP, only in nvHABP do all these values reach statistical significance across all organisms. This suggests that future quality efforts need to address this syndrome specifically as it represents an area where marginal improvements in diagnostics and pathogen identification may result in significant, patient centered improvements.

Although earlier studies have shown consistent worsening of hospital outcomes, including mortality, LOS, and costs, in association with IET, this was in the setting of much higher rates of IET. For example, Alvarez-Lerma et al. reported an IET rate of 27% in a cohort of 490 patients enrolled during the late 1980s in Spain with an ICU-acquired pneumonia [16]. In a smaller single-center cohort study of patients with VABP in the US in the early 2000s, Iregui and coworkers found an IET rate of 31% [17]. Both these and other studies conducted in earlier eras have documented a high prevalence of IET and its detrimental effects to patients. A meta-analysis of studies examining the efficacy of non-IET in sepsis published in 2010 identified 70 suitable studies, of which 13 focused on pneumonia (9 ventilator-associated) [18]. The enrollment periods for these 13 investigations spanned 1988–2005, with three (all VABP) from 2000 or later, including Iregui et al. [17]. Similar to the US study, Seligman et al. reported the rate of IET in a Brazilian institution between 2003 and 2005 to be 27% [19]. In contrast, a study from France, 2001–2004, found a much lower rate of IET of 13% [20].

A more recent meta-analysis of hospital-initiated IET in severe infections reporting on 27 studies published between 2004 and 2014 found a wide range of IET between 14 and 79% [21]. In six papers with only pneumonia patients, IET was reported in 49–68% (52–68% HABP/VABP only). Notably, none of these studies enrolled after 2010, and none was set in the US [21]. A subsequent multicenter study from Korea in the setting of NP in 2019 reported that 44% of all HABP/VABP patients received IET [22]. This rate is most likely explained by the extremely high prevalence of GN infections (91%) and multidrug resistance (70%).

Our investigation not only provides the most current snapshot of microbiology of NP in the US, but also adds granularity to the interaction between organisms and empiric treatment, with a particular emphasis on pathogens that raise the risk of IET. While IET is lower in prevalence than previously reported, < 10%, across all three types of NP examined, there is considerable variation by organism: highly resistant pathogens are rare, but highly likely to undergo IET. This concurrence of IET with the infrequent isolation of highly drug resistant organisms explains the relative low prevalence of IET in this broadly defined cohort. This suggests that treatment paradigms that recommend initiating therapy with broad spectrum agents have generally proven successful at diminishing rates of IET, but have, nonetheless, left gaps in selected scenarios.

We further confirm that at least some of the outcomes continue to be worse in the setting of IET, though not all, and not across all pneumonia types. It bears mentioning that in some cases, such as in VABP when it is due to a GP or a polymicrobial mixed GP and a GN etiology, hospital mortality paradoxically trends higher with non-IET than with IET. There are two potential explanations for this. First, since these differences did not reach statistical significance, it may be an artifact of small sample size. On the other hand, it is also possible that with greater power, the difference might have been significant. If so, this contradictory result would most likely be due to a confounding by indication, whereby a sicker patient, as discerned by the clinician at the bedside (as opposed to a metric derived from a database), is more likely to have an adverse outcome despite his/her higher propensity to receive broader spectrum empiric regimen.

What are the clinical implications of our findings? The heterogeneity of interactions of IET with organism and pneumonia types suggests that more accurate targeting of empiric treatment may not appreciably improve outcomes across the board. Rather, it may be more prudent to expend more energy on preventing this hospital-acquired infection. To reduce rates of IET further may necessitate using overly broad agents to cover very rare pathogens. However, embracing this strategy also carries risk, and may promote further AMR with little potential benefit to institutions or society. When, despite best efforts at prevention a breakthrough case occurs, the risk of a GN pathogen specifically in the setting of nvHABP should be evaluated carefully and broad treatment instituted when such risk is deemed high enough. At the bedside, in the absence of rapid molecular testing, our data reinforce the common recommendation to rely on clinical risk factors alongside the current antibiogram when making treatment decisions.

Our study has a number of strengths and limitations. As a large geographically representative multicenter cohort, its results are highly generalizable to bacterial NP treated in US institutions. The presence of microbiology data, as well as timed administration of medications and other treatments, allow for a more accurate definition of bacterial NP and exploration of therapies aimed at it. Furthermore, excluding other potential infections, such as cIAI and cUTI prevent misclassifying them as NP. One aspect of how we defined microbiological infection bears emphasis. Since we required either a respiratory or a blood culture, it is possible that when both were positive in a single patient, this represented not pneumonia, but rather a concurrence of pneumonia and a blood stream infection unrelated to pneumonia, possible a catheter-related one. Since we did not exclude such cases, there is a possibility that we overestimated the prevalence of polymicrobial NP, and hence the actual rate of IET specific to NP may be even lower. Conversely, this increase in specificity necessarily reduces the sensitivity of our case definition. Furthermore, our interest in NP where a bacterial pathogen was identified narrows the applicability of our findings only to the minority of NP populations whose pathogen is isolated. Because post-discharge data were unavailable, our results apply only to events in the hospital. Furthermore, this limitation impacts the estimate of 30-day readmissions in two possible ways. First, the denominator for this calculation may be inflated, as we did not have access to out-of-hospital mortality. Second, only readmissions to the same hospital in the Premier database are trackable, resulting in the potential to underestimate the volume of such events. Overall, however, these omissions are present irrespective of whether the index hospitalization included IET or not. Bias, a common problem with observational studies, is a potential issue here as well. We have tried to mitigate selection bias by setting a priori case definitions and enrolling all consecutive patients based on these criteria. While confounding may reduce the strength of causal inference, our study limited itself to descriptions of associations.

## Conclusions

In summary, we have demonstrated that in the setting of bacterial NP, the prevalence of IET is lower than reported in the past. While it impacts hospital outcomes differentially depending on both the type of NP and the organism, in many instances it is associated with numerically higher mortality and costs. Fortunately, highly resistant organisms, which have the highest likelihood of predisposing to IET receipt, remain rare. Our data also suggest that, while the best outcome is NP prevention, in the event one develops, different types of and pathogens in NP demand variable levels of coverage deployment. Namely, hospitals with high prevalence of GN pathogens need to pay more attention to targeting treatment more broadly than those with higher likelihood of GP. Such broader approach to empiric therapy, however, should not be the end of targeted decision making. Indeed, the broader the empiric choice, the more important it becomes to attend to prompt de-escalation once the organism has been identified. In the absence of such de-escalation, we are simply postponing the inevitable transformation of the current poor individual outcomes associated with IET into the global catastrophe of the end of antibiotics due to the loss of their activity in AMR.

## Supplementary Information

**Additional file 1: Table S1.** Infection characteristics and inappropriate empiric treatment.

**Additional file 2: Table S2.** Pathogens most commonly accounting for IET exposure.

## Data Availability

The data used in this study derive from Premier Healthcare Database, a proprietary third-party database available to researchers through a specific agreement with Premier.

## Abbreviations

NP: Nosocomial pneumonia
nvHABP: Non-ventilated hospital-acquired bacterial pneumonia
vHABP: Ventilated hospital-acquired bacterial pneumonia
VABP: Ventilator-associated bacterial pneumonia
MV: Mechanical ventilation
MRSA: Methicillin-resistant *S. aureus*
MSSA: Methicillin-susceptible *S. aureus*
IET: Inappropriate empiric therapy
AMR: Antimicrobial resistance
LOS: Length of stay
S: Susceptible
R: Resistant
I: Intermediate
CR: Carbapenem resistant
CS: Carbapenem susceptible
C3R: Non-susceptible to third generation cephalosporins
ESBL: Extended spectrum beta-lactamase
SD: Standard deviation
IQR: Interquartile range
GN: Gram-negative
GP: Gram-positive
