# Supplementary material for: The risk of inappropriate empiric treatment and its outcomes based on pathogens in non-ventilated (nvHABP), ventilated (vHABP) hospital-acquired and ventilator-associated (VABP) bacterial pneumonia in the US, 2012–2019
Source: BMC Infect Dis. 2022 Oct 5;22:775. doi: 10.1186/s12879-022-07755-y (PMC9533487; doi:10.1186/s12879-022-07755-y)
Supplement: Supplementary file 2 — Additional file 2: Table S2. Pathogens most commonly accounting for IET exposure. [file 12879_2022_7755_MOESM2_ESM.docx]

**Supplemental Table 2. Pathogens most commonly accounting for IET exposure**

| **Pathogen** | **nvHABP** | **vHABP** | **VABP** |
| --- | --- | --- | --- |
| *Pseudomonas aeruginosa* | 24.18% | 17.37% | 18.51% |
| *Escherichia coli* | 11.97% | 14.67% | 5.54% |
| *MSSA* | 11.74% | 12.74% | 12.34% |
| *MRSA* | 11.50% | 8.88% | 6.33% |
| *Klebsiella pneumoniae* | 11.03% | 8.11% | 9.02% |
| *Enterobacter cloacae* | 4.93% | 7.72% | 9.34% |
| *Stenotrophomonas maltophilia* | 3.76% | 8.49% | 10.13% |
| *Serratia marcescens* | 3.52% | 4.25% | 6.65% |
| *Proteus mirabilis* | 2.82% | 0.39% | 1.11% |
| *Acinetobacter baumannii* | 2.82% | 6.56% | 6.80% |
| *Haemophilus influenzae* | 2.82% | 1.16% | 3.16% |
| *Enterobacter aerogenes* | 2.58% | 3.09% | 2.85% |
| *Klebsiella oxytoca* | 2.11% | 1.54% | 2.22% |
| *Streptococcus pneumoniae* | 2.11% | 2.32% | 2.53% |
| *Providencia spp* | 0.47% | 0.39% | 0.32% |
| *Citrobacter freundii* | 0.47% | 0.77% | 0.32% |
| *Citrobacter other* | 0.47% | 0.77% | 0.47% |
| *Streptococcus other* | 0.47% | 0.00% | 0.32% |
| *Morganella morganii* | 0.23% | 0.39% | 0.47% |
| *Proteus other* | 0.00% | 0.00% | 0.32% |
| *Enterobacter other* | 0.00% | 0.39% | 0.32% |
| *Serratia other* | 0.00% | 0.00% | 0.79% |
| *Klebsiella other* | 0.00% | 0.00% | 0.16% |
| *Moraxella catarrhalis* | 0.00% | 0.00% | 0.00% |

IET = inappropriate empiric treatment; MSSA = methicillin-susceptible *S. aureus*; MRSA = methicillin-resistant *S. aureus*; nvHABP = non-ventilated hospital-acquired bacterial pneumonia; vHABP = ventilated hospital-acquired bacterial pneumonia; VABP = ventilated hospital-acquired bacterial pneumonia
